# Neutralizing RGMa with Elezanumab Promotes Cerebroprotection and Recovery in Rabbit Middle Cerebral Artery Occlusion

**DOI:** 10.1007/s12975-023-01164-2

**Published:** 2023-06-16

**Authors:** Peer B. Jacobson, Andrea Mothe, Aharon Levy, Michael Krakovsky, Bradley A. Hooker, Xiaomeng Zhang, Jennifer Mollon, Yulia Mordashova, Mathias Droescher, Sabine Weiss, Stefan Barghorn, Ingeborg Dreher, Khader Awwad, Volker Nimmrich, Lili Huang, Emma Fung, Wayne R. Buck, Kimberly Pfleeger, Adam Ziemann, Elaine Smith, Gerard B. Fox, Charles H. Tator, Michael Gold

**Affiliations:** 1 Department of Translational Sciences, Imaging Research, AbbVie Inc., 1 North Waukegan Rd, North Chicago, IL 60064 USA; 2 Division of Experimental and Translational Neuroscience, Krembil Brain Institute & University Health Network, Toronto, ON M5T 0S8 Canada; 3 Pharmaseed Ltd, 74047 Ness Ziona, Israel; 4 Data and Statistical Sciences, AbbVie Deutschland GmbH & Co. KG, Neuroscience Research, 67061 KnollstrasseLudwigshafen, Germany; 5 Discovery Biology, AbbVie Deutschland GmbH & Co. KG, Neuroscience Research, 67061 Knollstrasse, Ludwigshafen Germany; 6 Department of Drug Metabolism, Pharmacokinetics and Bioanalysis, AbbVie Deutschland GmbH & Co. KG, 67061 Knollstrasse, Ludwigshafen Germany; 7 AbbVie Biologics, AbbVie Bioresearch Center, 100 Research Drive, Worcester, MA 01605 USA; 8 Preclinical Safety, AbbVie Inc, 1 North Waukegan Rd, North Chicago, IL 60064 USA; 9 Department of Neuroscience Development, AbbVie Inc, 1 North Waukegan Rd, North Chicago, IL 60064 USA; 10 Division of Experimental and Translational Neuroscience, Krembil Brain Institute & University Health Network, Toronto, ON M5T 0S8 Canada; 11 Division of Neurosurgery, Department of Surgery, University of Toronto, Toronto, ON M5T 2S8 Canada

**Keywords:** Elezanumab, Acute ischemic stroke, MCAO, Rabbit, RGMa, Cerebroprotection

## Abstract

Repulsive guidance molecule A (RGMa) is an inhibitor of neuronal growth and survival which is upregulated in the damaged central nervous system following acute spinal cord injury (SCI), traumatic brain injury, acute ischemic stroke (AIS), and other neuropathological conditions. Neutralization of RGMa is neuroprotective and promotes neuroplasticity in several preclinical models of neurodegeneration and injury including multiple sclerosis, AIS, and SCI. Given the limitations of current treatments for AIS due to narrow time windows to intervention (TTI), and restrictive patient selection criteria, there is significant unmet need for therapeutic agents that enable tissue survival and repair following acute ischemic damage for a broader population of stroke patients. In this preclinical study, we evaluated whether elezanumab, a human anti-RGMa monoclonal antibody, could improve neuromotor function and modulate neuroinflammatory cell activation following AIS with delayed intervention times up to 24 h using a rabbit embolic permanent middle cerebral artery occlusion model (pMCAO). In two replicate 28-day pMCAO studies, weekly intravenous infusions of elezanumab, over a range of doses and TTIs of 6 and 24 h after stroke, significantly improved neuromotor function in both pMCAO studies when first administered 6 h after stroke. All elezanumab treatment groups, including the 24 h TTI group, had significantly less neuroinflammation as assessed by microglial and astrocyte activation. The novel mechanism of action and potential for expanding TTI in human AIS make elezanumab distinct from current acute reperfusion therapies, and support evaluation in clinical trials of acute CNS damage to determine optimal dose and TTI in humans.

**Graphical Abstract:**

A: Ramified/resting astrocytes and microglia in a normal, uninjured rabbit brain. B: Rabbit pMCAO brain illustrating lesion on right side of brain (red), surrounded by penumbra (pink) during acute phase post stroke, with minimal injury to left brain hemisphere. Penumbra characterized by activated astrocytes and microglia (region in crosshair within circle), with upregulation of free and bound RGMa. C: Elezanumab binds to both free and bound RGMa, preventing full activation of astrocytes and microglia. D: Elezanumab is efficacious in rabbit pMCAO with a 4 × larger TTI window vs. tPA (6 vs. 1.5 h, respectively). In human AIS, tPA is approved for a TTI of 3-4.5 h. Elezanumab is currently being evaluated in a clinical Ph2 study of AIS to determine the optimal dose and TTI (NCT04309474).
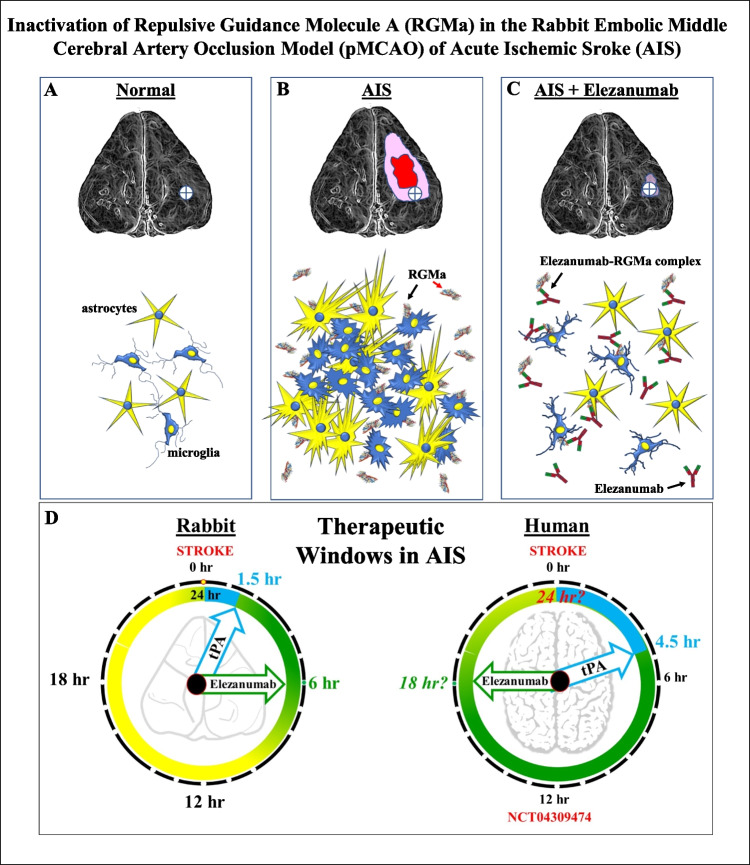

**Supplementary Information:**

The online version contains supplementary material available at 10.1007/s12975-023-01164-2.

## Introduction

Despite public awareness campaigns and improvements in stroke quality of care, stroke remains the second leading global cause of death and disability [1, 2]. A total of 12.2 million cases of stroke were documented in 2019, with an annual mortality rate of nearly 5.5 million; 87% of cases were attributed to acute ischemic stroke (AIS) [1]. The burden of ischemic stroke is projected to increase due to aging populations with multiple comorbidities, and new strategies to mitigate the damage caused by this devastating condition are urgently needed [3].

The impact of ischemic stroke is further aggravated due to limited acute therapeutic options. The only widely approved pharmacologic therapy for AIS is tissue-type plasminogen activator (tPA; Alteplase®, Activase®, Actilyse®), which is approved for use within 3 h since known onset of stroke symptoms [4], although many national guidelines allow for administration of tPA up to 4.5 h after stroke onset [5]. The only other available acute intervention is endovascular therapy (EVT), which is specifically targeted for patients with large vessel occlusions. EVT must be initiated within 6 h of stroke onset for patients with eligible symptoms and occlusion location, but may be considered up to 24 h after stroke for selected patients with advanced imaging confirming the presence of salvageable tissue [6].

Repulsive guidance molecule a (RGMa) is a glycosylphosphatidylinositol anchored glycoprotein that exists in membrane-bound and soluble forms [7, 8]. RGMa binds to the receptor neogenin, and acts as a coreceptor for bone morphogenic proteins (BMPs), subsequently activating downstream pathways leading to apoptosis, demyelination, and inhibition of axonal regeneration. In the adult CNS, RGMa helps maintain the integrity of neural networks and regulates neuroplasticity [9]. In pathological conditions, including ischemia, RGMa is aberrantly upregulated in the CNS at sites of injury or inflammation, where it contributes to demyelination, cell death, increased permeability of the blood–brain barrier, and neurite growth inhibition [10-12]. Elevated RGMa expression has been documented in the postmortem brains of human ischemic stroke victims as early as 12 h after stroke, and as long as 5 years after stroke [10].

Disruption of RGMa signaling using molecular and biological approaches results in decreased apoptosis and enhanced axonal growth in a variety of animal models of CNS injury [13]. Previous research has demonstrated the efficacy of several anti-RGMa interventions, including elezanumab, a human anti-RGMa monoclonal antibody, in preclinical models of acute spinal cord injury [14-16]. Elezanumab and its parental antibody (AE12-1Y) have also demonstrated neuroprotective and plasticity effects in ischemic and neuroinflammatory models including targeted experimental autoimmune encephalomyelitis, optic neuritis, and embolic stroke in the rat [8, 13].

In the present studies, we expanded our evaluation of RGMa neutralization with elezanumab in the rabbit pMCAO model in accordance with recommendations from STAIR and other expert panels which encourage the use of this model as an important step for translational validation [17, 18]. Although there are species differences [19], the neuromotor deficits and accompanying pathology in the rabbit pMCAO model demonstrate many correlations to human AIS [19], making the model relevant during the characterization of tPA, the only approved drug for AIS [5, 20]. Here, we found that initial administration of elezanumab 6 h after pMCAO induced improvements in neuromotor function and attenuation of glial reactivity. Elezanumab’s unique mechanism of action, combined with its extended TTI in this preclinical model, supports its potential to benefit a broader population of patients than current therapy. Elezanumab is currently being evaluated in Ph2 clinical studies for acute spinal cord injury (NCT04295538) and acute ischemic stroke (NCT04309474).

## Methods

### Antibody Characterization, Formulation, and Quantification

Characterization of the human anti-RGMa antibody elezanumab (also known as AE12-1Y-QL or ABT-555; AbbVie, North Chicago, IL) has been previously reported [8]. Analytic methods used to quantify elezanumab and the control IgG antibodies are described in detail in the Supplementary Information.

### Animal Welfare

Animal handling followed the guidelines of the National Institute of Health (NIH) and the Association for Assessment and Accreditation of Laboratory Animal Care (AAALAC). Pharmaseed preclinical studies were conducted at Ness Ziona, Israel. Additional accreditation details are provided in the Supplementary Information.

### Animal Housing

Male New Zealand White rabbits weighing 2.8–3.6 kg (15–18 weeks of age) at study initiation were sourced from Envigo RMS Ltd., Israel, and housed under standard laboratory conditions detailed in the Supplementary Information.

### Rationale for Test Group Size

The number of groups and the total number of rabbits per group were based upon previous studies demonstrating a sample size of ~ 10/group as the minimal number of animals per group sufficient to obtain reliable information. Each experimental group included 10–13 animals to yield at least 8–10 surviving animals at study termination.

### Rabbit pMCAO Surgical Procedure

The occlusion of the middle cerebral artery followed a modified procedure described by Zhao et al. [21], and is described in detail in the Supplementary Information. In brief, following exposure of the right external carotid artery (ECA), a homologous blood clot was injected into the cerebral circulation via the ECA, and occlusion of cerebral blood flow was confirmed in each animal using transcranial laser Doppler.

### Study Designs

#### *Study 1*

Surgeries in both studies were performed by a single, trained surgeon. Six hours after pMCAO surgery, animals were randomly assigned to one of 3 treatment groups based on post pMCAO transcranial laser Doppler measurements of cerebral blood flow: (1) IgG control antibody (80 mg/kg IV first dose, followed by 3 weekly IV doses of 40 mg/kg); (2) elezanumab (20 mg/kg IV first dose, followed by 3 weekly IV doses of 10 mg/kg); and (3) elezanumab (80 mg/kg IV first dose, followed by 3 weekly IV doses of 40 mg/kg); final group sizes ranged between 11 and 13/group. Neurological scoring was first performed 48 h after pMCAO, and on days 7, 14, 21, and 28 by a single trained observer blinded to treatment. Following each scoring session, written scoring results were securely maintained until the end of the study, minimizing unintended bias from previous scoring sessions. At study termination on day 28, animals were perfused intracardially with cold, heparinized saline followed by at least ten blood volumes of cold, 4% buffered paraformaldehyde. Brains were harvested and fixed for 24 h, then transferred into sterile phosphate-buffered saline containing 0.01% sodium azide for storage at 4ºC.

#### *Study 2*

Six hours after pMCAO surgery, animals were randomly assigned to one of the following 4 treatment groups: (1) IgG control (20 mg/kg first dose 6 h, followed by 3 weekly IV doses of 10 mg/kg); (2) elezanumab (2 mg/kg first dose 6 h, followed by 3 weekly IV doses of 1 mg/kg); (3) elezanumab (20 mg/kg first dose 6 h, followed by 3 weekly IV doses of 10 mg/kg); and (4) elezanumab (20 mg/kg first dose 24 h, followed by 3 weekly IV doses of 10 mg/kg). Final group sizes were 8–10 animals/group. Neurological scoring was performed 24 h after pMCAO and on days 7, 14, 21, and 28 by the same experienced observer who performed assessments in study 1. Blinding and data handling were conducted in an identical manner as in study 1. At study termination, animals were intracardially perfused as previously described.

### Morbidity and Mortality

Detailed in the Supplementary Information.

### Neuromotor Scoring

Neuromotor recovery was evaluated using a composite score comprising a comprehensive array of clinical-neurological tests including motor, sensory, reflex, and balance [22]. Neuromotor scoring was graded on a scale of 0 to 9 (where a normal animal was scored 0 and an animal with maximal deficits was assigned a score of 9). Descriptions of each test, and scoring criteria, are listed in Table 1.

**Table 1:** Rabbit pMCAO neuromotor assessments

Neurological test	Neurological assessment	Score (0 = normal; 9 = max deficit)
Wry-neck test	Torsion of the neck; shoulder adduction	0 = normal 1 = twisting of neck
Righting reflex	Animal placed on back, and its ability to right itself is observed	0 = within 1 s; 1 = within 5 s; 2 ≥ 5 s
Paw dysfunction	Ability to re-extend paw when pulled toward body	0 = within 1 s; 1 = within 5 s 2 ≥ 5 s
Postural reflex	Animal is pushed in contralateral direction to assess resistance	0 = normal 1 = reduced resistance to lateral push; 2 = falls down on contralateral side
Circling test	Observe if animal moves in a circle when body is touched	0 = normal; 1 = animal circles after touching its body; 2 = if animal circles without touching its body

### Imaging

Ex vivo images from fixed brains in study 2 were acquired at 7 Tesla using a BioSpec 70/30 MRI system (Bruker, Billerica, MA) with a B-GA 12SL gradient and shim insert and a 23-mm transceiver volume coil. Methodology and sequence details are provided in the Supplementary Information and shown in Figure S1.

### Histopathology

Following ex vivo imaging, paraformaldehyde-fixed rabbit brains were processed at NeuroScience Associates (Knoxville, TN). Blocks were cut at 40 µm through the entire specimen segment and slices collected sequentially. Each section is approximately 1.4 mm apart from its adjacent section. Methods and antibodies used for immunostaining are detailed in the Supplementary Information. For analysis, approximately 20 consecutive Iba-1 and GFAP sections per animal were digitized and evaluated in ImageJ after establishing individual thresholding levels for each stain using somas from non-activated cells in the contralateral/left hemisphere (immunostained cells marked in red; Figure S2). Red pixels within the section were then quantified and expressed as a percentage of total tissue area. Animal identification numbers (IDs) were blinded and randomized across treatment groups prior to stereological analysis by a single trained investigator.

### Statistics

Differences between treatment groups were assessed using Dunnett’s test. When there was evidence of non-homogeneity of variances, skewed data were log-transformed before testing. All *p*-values are uncorrected for multiplicity except for those from the Dunnett tests. In addition to Dunnett tests for multiple comparisons, analysis of Iba-1 data included the Benjamini–Hochberg procedure to generate *q* values which are controlled for false discovery rates. JMP 14.1.0 (SAS Institute), R studio Version 1.1.453 with R V4.0.5, and GraphPad Prism V.9.1.0 (GraphPad Software, Inc., San Diego, CA) were used for statistical analysis and data presentation.

## Results

### Elezanumab Pharmacokinetics in Rabbits

**Fig. 1:**
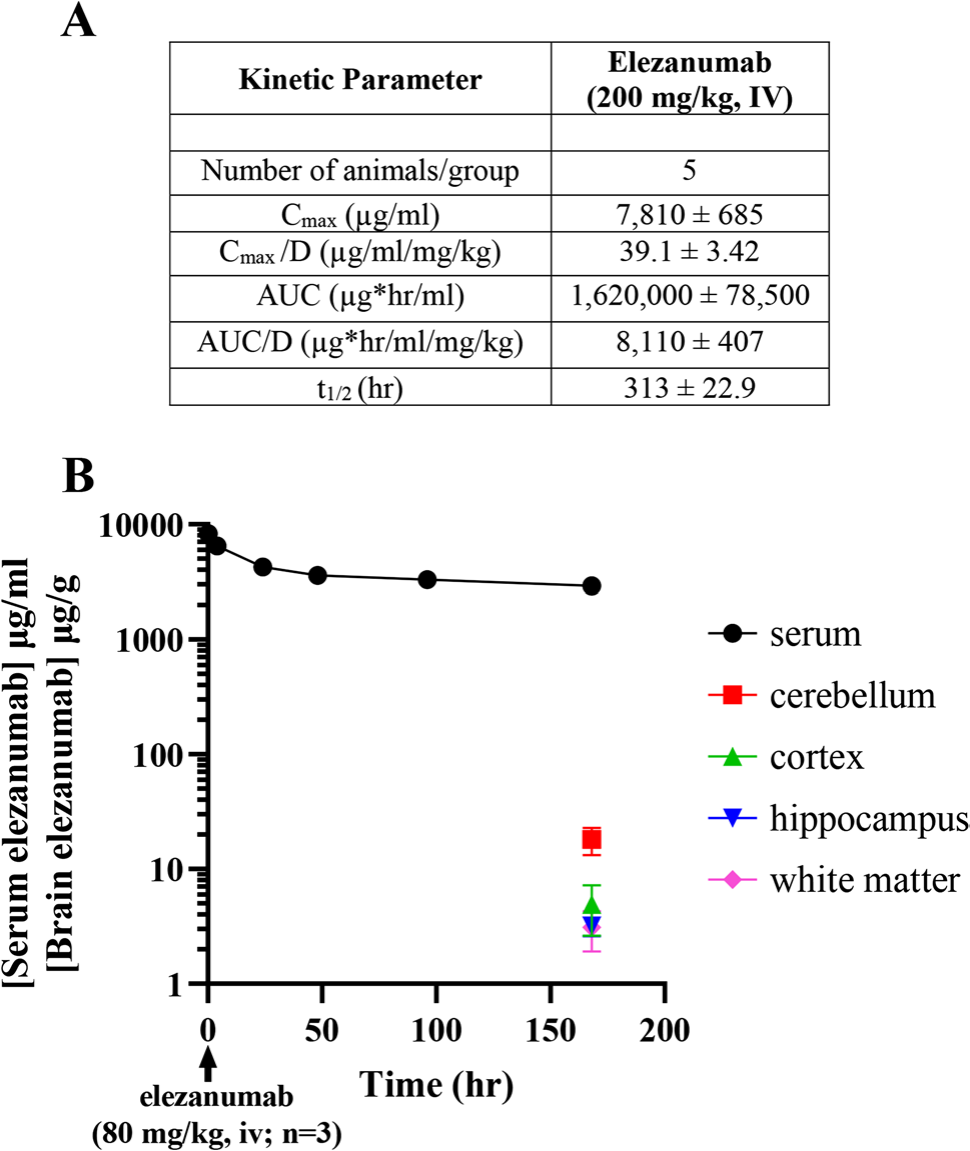
Pharmacokinetics of elezanumab in normal white New Zealand male rabbits. **A** Kinetic parameters following a single 200 mg/kg, IV dose of elezanumab (*n* = 5). **B** Serum concentration of elezanumab in rabbits (*n* = 3) over 7 days and terminal tissue concentrations following a single 80 mg/kg, IV dose

Prior to initiating efficacy studies in the rabbit pMCAO model, the pharmacokinetics of elezanumab were established in uninjured rabbits. As shown in Fig. 1A, the mean terminal half-life of elezanumab was 313 ± 22.9 h (calculated as a harmonic mean ± standard deviation) following a single 200 mg/kg, IV dose. In a second pharmacokinetic study, longitudinal serum concentrations of elezanumab and terminal brain tissue concentrations were determined over 7 days following a single 80 mg/kg IV dose in uninjured rabbits (Fig. 1B). Serum levels on day 7 were 2.921 ± 217 µg/ml, with brain concentrations of 18.1 ± 4.8 µg/g in the cerebellum (0.62% of [serum]), 4.9 ± 2.3 µg/g in cortical tissue (0.17% [serum]), 3.19 ± 0.58 µg/g in hippocampus (0.11% of [serum]), and 3.10 ± 1.89 µg/g in white matter (0.11% of [serum]).

### Efficacy of Elezanumab in Improving Functional Recovery in Rabbit pMCAO

Elezanumab was evaluated in two replicate, randomized, observer-blinded studies using the rabbit pMCAO model over a range of doses and initial administration times to assess neuromotor function and other biochemical, imaging, and histological endpoints.

#### Efficacy Study 1

**Fig. 2:**
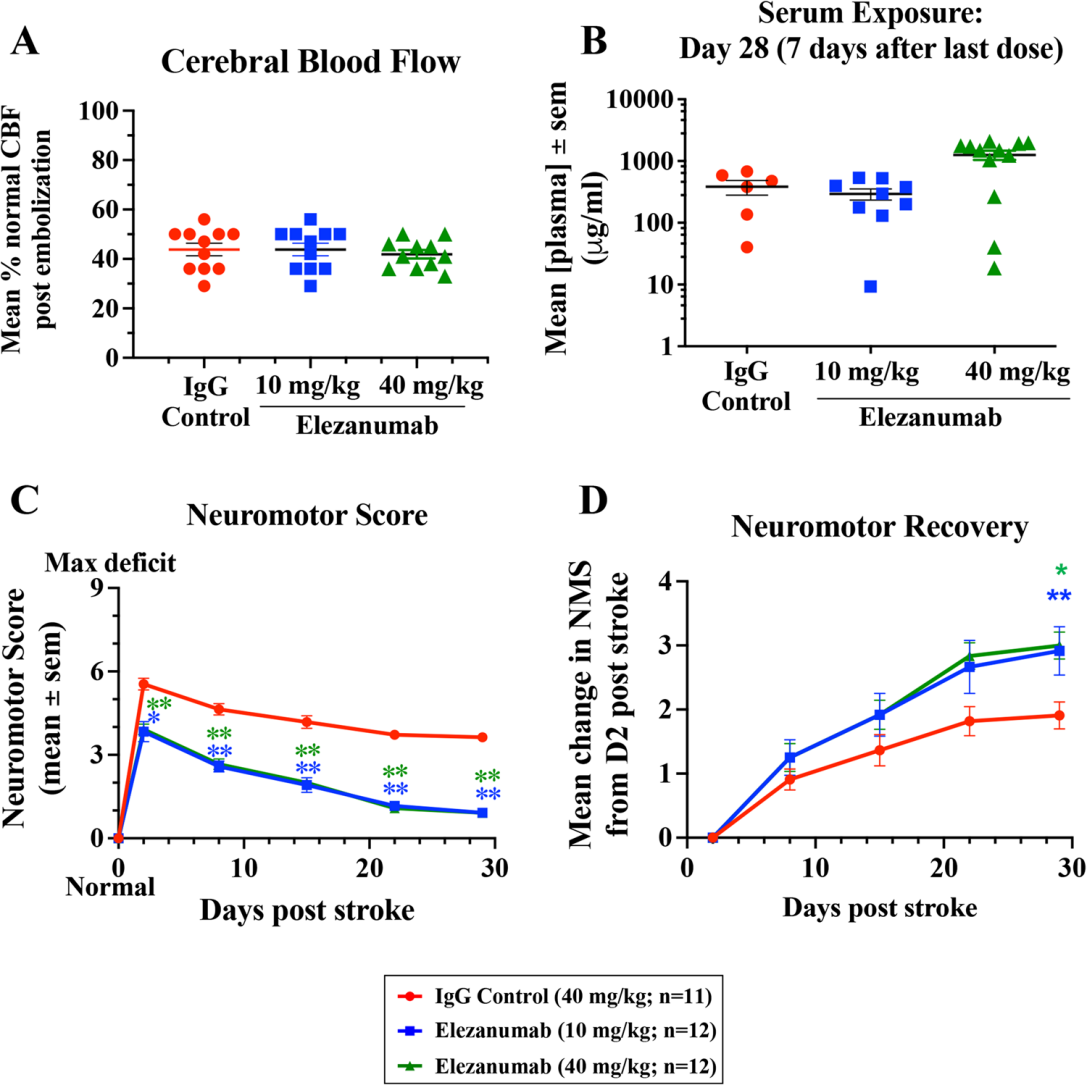
Rabbit study 1. **A** CBF before and after occlusion; data are expressed as the % of pre-MCAO CBF. **B** Mean serum levels of elezanumab at the end of the study, 7 days after the last IV injection. **C** Average neuromotor scores over the 28-day study where * *p* < 0.001 and ** *p* < 0.0001 using Fischer’s exact test. Recovery in these groups continued to increase over the 28-day study, with both elezanumab treatment groups demonstrating significant effects in the recovery rate (panel **D**; 1 mg/kg, 6 h FD group, *p* = 0.002 vs. control; 10 mg/kg, 6 h FD group, *p* = 0.03 vs. control, both using the one-sided Mann–Whitney *U* test)

Cerebral blood flow (CBF) values immediately following pMCAO (prior to any treatment intervention) were reduced by 58% of pre-occlusion values across the three study groups, indicating consistent levels of injury in all animals (Fig. 2A). At the 28-day endpoint, elezanumab exposure was dose-dependent (10 mg/kg: 295 ± 60 µg/ml; 40 mg/kg: 1,258 ± 219 µg/ml). Two animals in the IgG control group, one in the 10 mg/kg group, and 3 in the 40 mg/kg elezanumab group had lower exposures at the end of the study, possibly due to the presence of anti-drug antibodies (ADAs), although no further tests were initiated to confirm the presence of ADAs (Fig. 2B).

Early and sustained treatment effects were observed with both doses of elezanumab compared with control. Both doses of elezanumab, administered 6 h post pMCAO, significantly reduced peak neuromotor deficits 48 h post stroke (control: 5.55 ± 0.21; elezanumab 10 mg/kg: 3.83 ± 0.37 (*p* < 0.05 vs. control); elezanumab 40 mg/kg: 3.92 ± 0.19 (*p* < 0.01 vs. control); Fig. 2C). Over the 28-day time course, both the 10 and 40 mg/kg doses of elezanumab demonstrated a nearly identical efficacy response vs. IgG controls (day 28 NMS: control, 3.63 ± 0.15; elezanumab, 10 mg/kg, 0.92 ± 0.08 (*p* < 0.01 vs. control); elezanumab, 40 mg/kg, 0.92 ± 0.08 (*p* < 0.01 vs. control)), with a roughly 3 × greater rate of recovery during the study vs. controls (control: 0.03 neuromotor units (NMU)/day) vs. 10 mg/kg and 40 mg/kg elezanumab: 0.10 NMU/day (*p* < 0.01 and *p* < 0.05 vs. control, respectively; Fig. 2D).

#### Efficacy Study 2

**Fig. 3:**
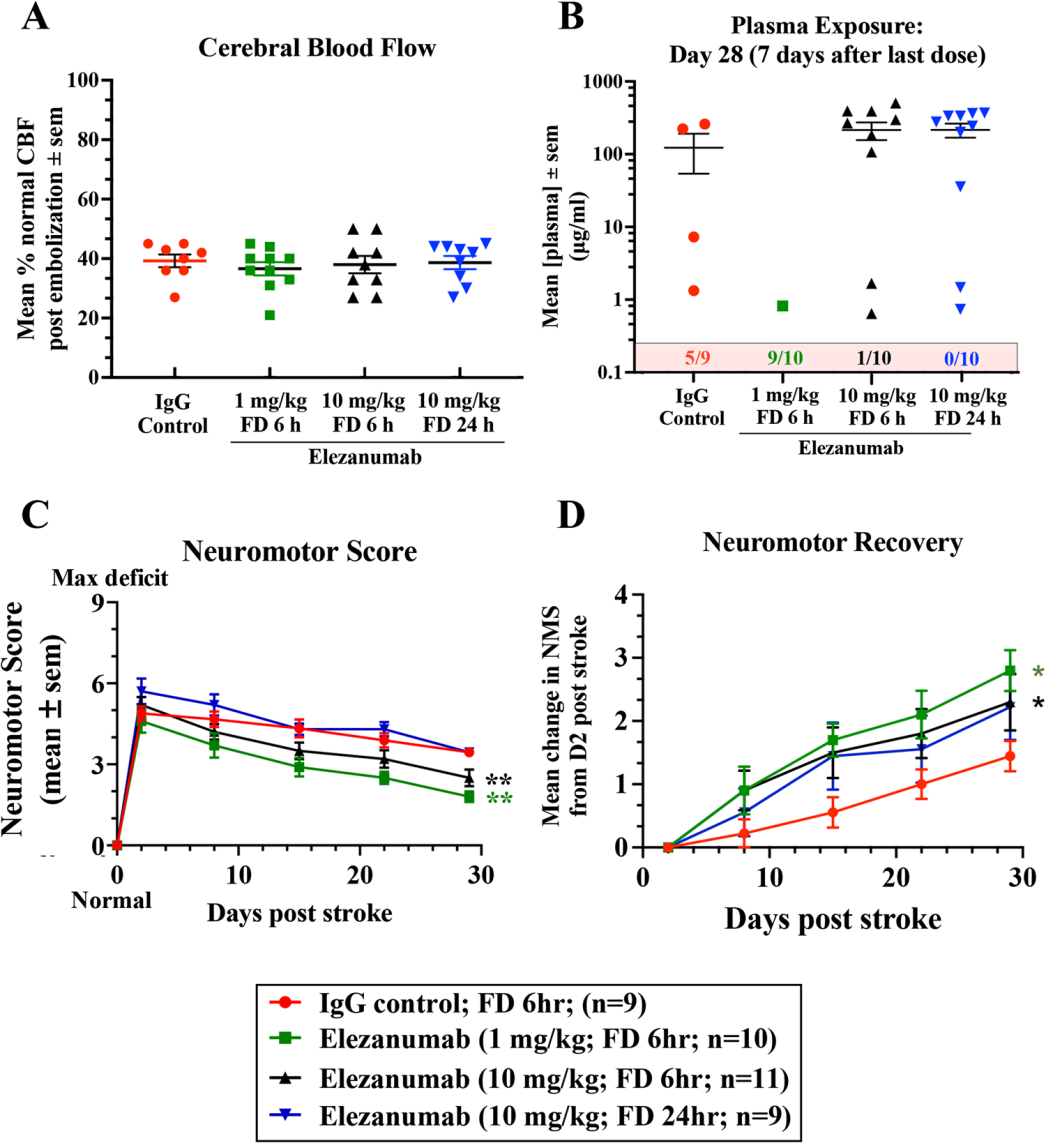
Rabbit study 2. **A** CBF before and after occlusion; data are expressed as the % of pre-pMCAO CBF. **B** Mean plasma exposures for IgG and elezanumab at the end of the study, 7 days after the last IV injection. Numbers in the pink box of panel B indicate the number of animals/group whose plasma drug levels were less than the lower limit of quantification. **C** Average neuromotor scores over the 28-day study. Data normalized to day 2 show all 6 h FD groups had a significantly greater rate of recovery vs. controls (**D**: 1 and 10 mg/kg, 6 h FD (*p* = 0.005 and *p* = 0.006, respectively), using the one-sided Mann–Whitney *U* test). The 10 mg/kg, 24 h FD group had a greater recovery rate vs. controls from day 2, but the difference was not significant (*p* = 0.16)

To confirm and expand upon these results, we conducted a second study, and in addition to repeating the 10 mg/kg dose, 6 h first dose, we evaluated a 10 × lower dose of elezanumab administered 6 h after stroke. We also examined the efficacy of 10 mg/kg elezanumab when the first administration was further delayed to 24 h after stroke. Similar to study 1, the reduction in CBF following pMCAO in rabbits did not significantly differ among groups, demonstrating consistent levels of injury among all animals (Fig. 3A). In study 2, the average reduction in CBF was approximately 60% in all groups immediately following pMCAO, which was similar to the reduction in CBF in study 1. Seven days after the last dose was administered on day 21, plasma IgG concentrations were 123 ± 69 µg/ml for controls, and elezanumab concentrations were 214 ± 58 µg/ml and 216 ± 47 µg/ml for the 10 mg/kg treated groups (6 and 24 h groups, respectively; Fig. 3B). Terminal plasma exposures for the 10 mg/kg, 6 h FD groups in study 1 vs. 2, were also comparable (study 1: 295 ± 60 µg/ml; study 2: 214 ± 58 µg/ml). Five of the 9 control animals, 9/10 of the 1 mg/kg elezanumab-treated animals, one animal in the 10 mg/kg, and 6 h group had IgG or elezanumab concentrations below the lower limits of quantification. Two animals from the 10 mg/kg, 6 h group, and 3 rabbits from the 10 mg/kg, 24 h FD group had measurable, but lower exposures by day 28, all of which suggest that some animals across groups may have developed ADAs by the end of the 28-day study.

Supporting findings from study 1, both doses of elezanumab administered at 6 h after pMCAO significantly enhanced recovery. Rabbits receiving an initial IV infusion of 1 or 10 mg/kg elezanumab 6 h after pMCAO showed significant improvements compared to IgG controls in both neuromotor function (*p* < 0.01 vs. controls; Fig. 3C) and rate of recovery (*p* < 0.05 vs. controls; Fig. 3D) by day 28. When the first 10 mg/kg elezanumab administration was delayed to 24 h after pMCAO, there were no significant differences in the neuromotor scores relative to controls (Fig. 3C), although rabbits showed a numerically faster recovery rate relative to controls (control: 1.44 ± 0.24; 10 mg/kg, 24 h FD: 2.22 ± 0.52) (Fig. 3D).

### Ex Vivo Magnetic Resonance and Diffusion Weighted Imaging

**Fig. 4:**
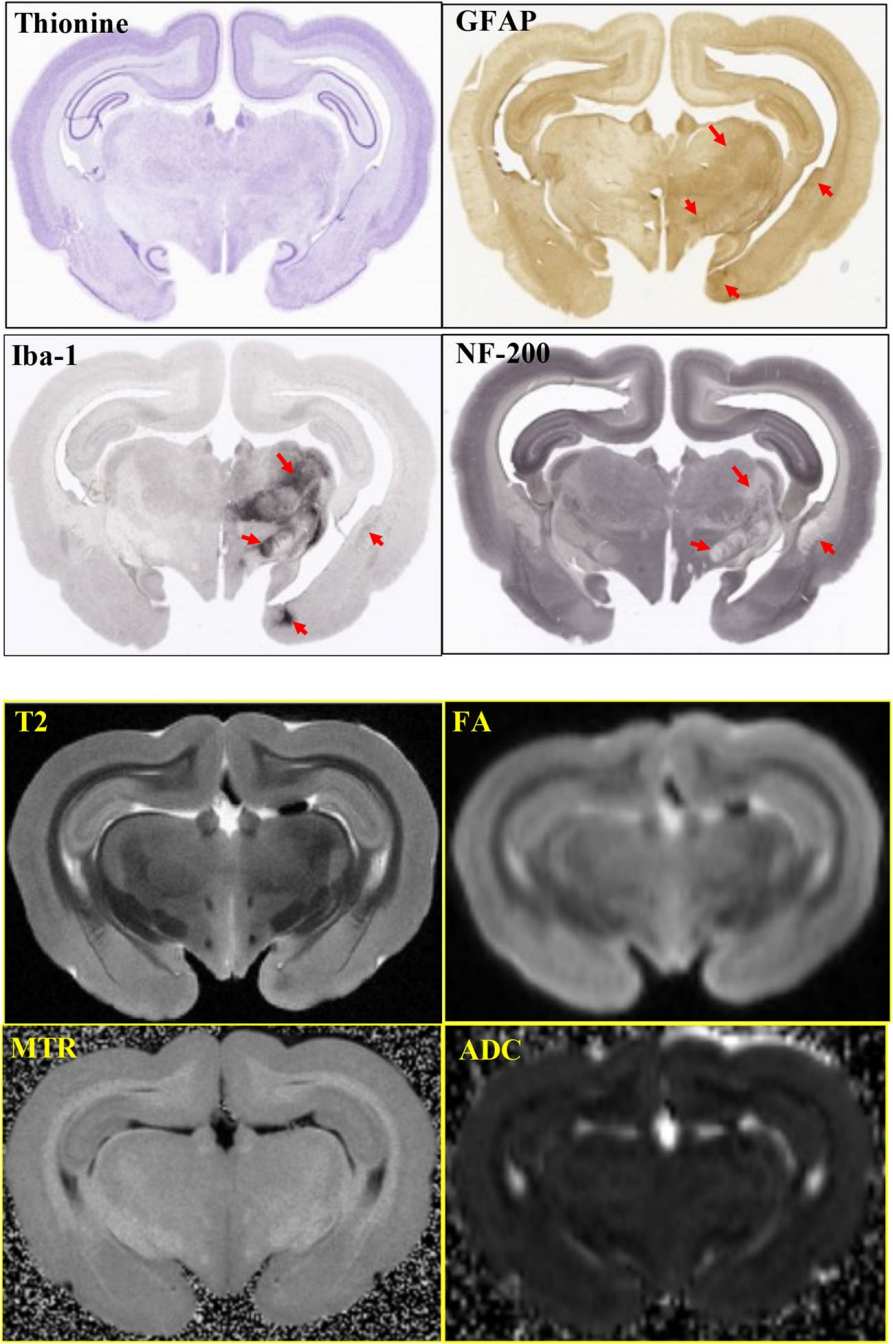
Histology and MRI/DTI. Adjacent 1.4-µm sections from a day 28 pMCAO control brain were evaluated using 4 stains including thionine, GFAP, Iba-1, and NF-200. Red arrows highlight corresponding regions of increased staining for GFAP and Iba-1, and the loss of NF-200 stain after 30 days following pMCAO. Higher magnification of thionine-stained tissues provided additional evidence of injury following stroke, and is reviewed in more detail in Figure S4. In contrast, ex vivo imaging of fixed intact brains taken 30 days after pMCAO typically shows minimal evidence of hypercellularity or lesions, with the exception of tissue undergoing pan-necrosis (see Figure S4 for more examples). Brain regions indicated by red arrows in the histology panels are challenging to identify in T2W images where few differences can be seen between “uninjured” left and injured right hemispheres

To examine apparent lesion locations and volumes prior to histopathology, fixed whole brains were initially imaged ex vivo using magnetic resonance and diffusion weighted imaging at endpoint (28 days post pMCAO). In Fig. 4, adjacent 40-µm sections from a single representative pMCAO control brain illustrate the sensitivities of four histological stains compared to their corresponding T2W/DTI images for identification of residual brain regions impacted by the stroke 28 days earlier. Hyperintense Iba-1 (microglia) and GFAP (astrocyte) staining along with a loss of NF-200 signal (neurofilament heavy chain) occur within the same regions in the injured right hemisphere (red arrows). However, T2W images and FA, MTR, and ADC parametric maps of the corresponding regions in the right hemisphere were in most cases indistinguishable from similar regions in the “uninjured” contralateral hemisphere, and could not be adequately segmented to quantify discrete lesion areas.

**Fig. 5:**
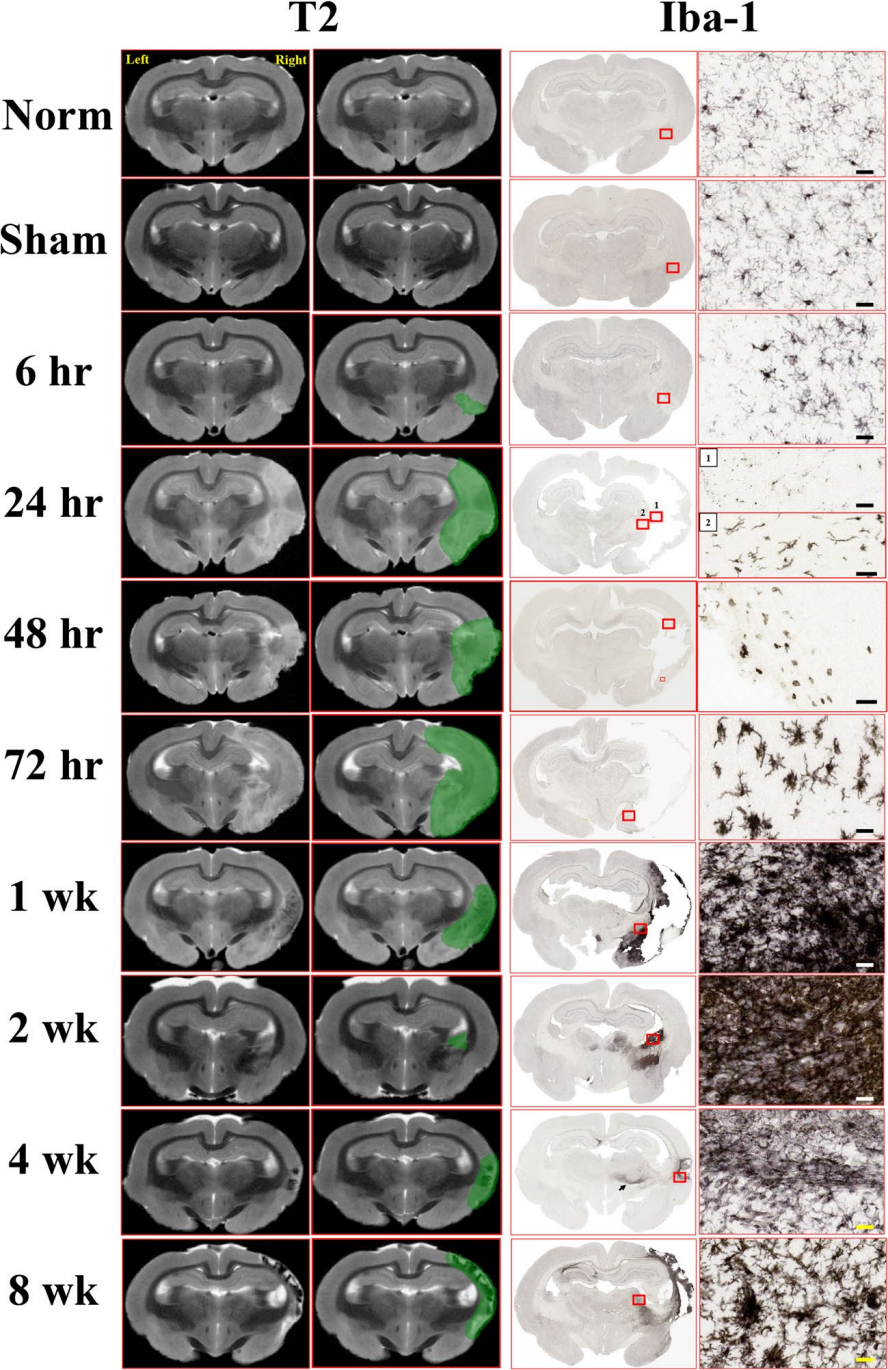
Longitudinal T2 imaging and microglial activation in the rabbit pMCAO model illustrating matched T2W images and Iba-1 stains from the same animal. The T2W panels on the left illustrate regions with hypo and hyperintensities; adjacent T2W panels have these areas masked in green. Regions of interest in the Iba-1 panels are identified with red boxes, and magnified in panels on the far right. Scale bars represent 50 µm. Full descriptions of these changes are described in the Supplemental Information

Longitudinal ex vivo imaging and Iba-1 histopathology associated with pMCAO in the rabbit reveal that edema and inflammatory processes peak ~ 72 h after the stroke (green masks; Fig. 5), coinciding with maximal neuromotor deficits (Figure S3A), peak plasma neurofilament levels, and other cellular changes (Figure S3A-E).

### Immunohistological Evidence of Elezanumab Effects on Gliosis

**Fig. 6:**
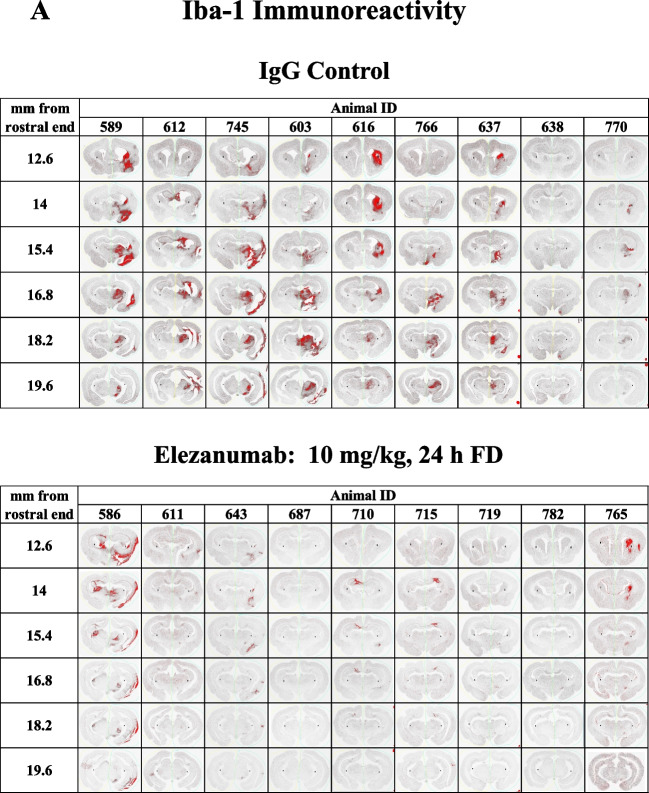

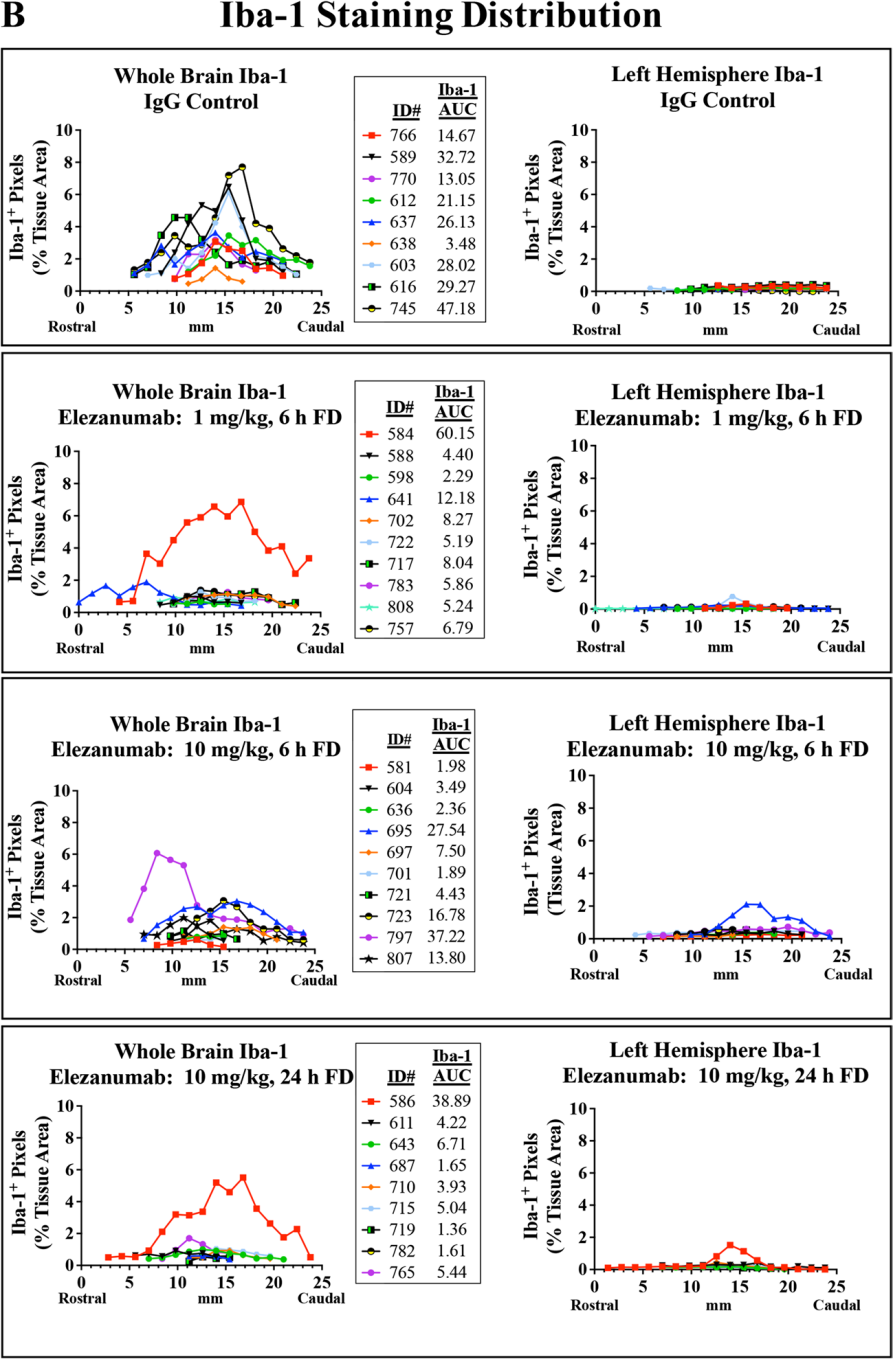

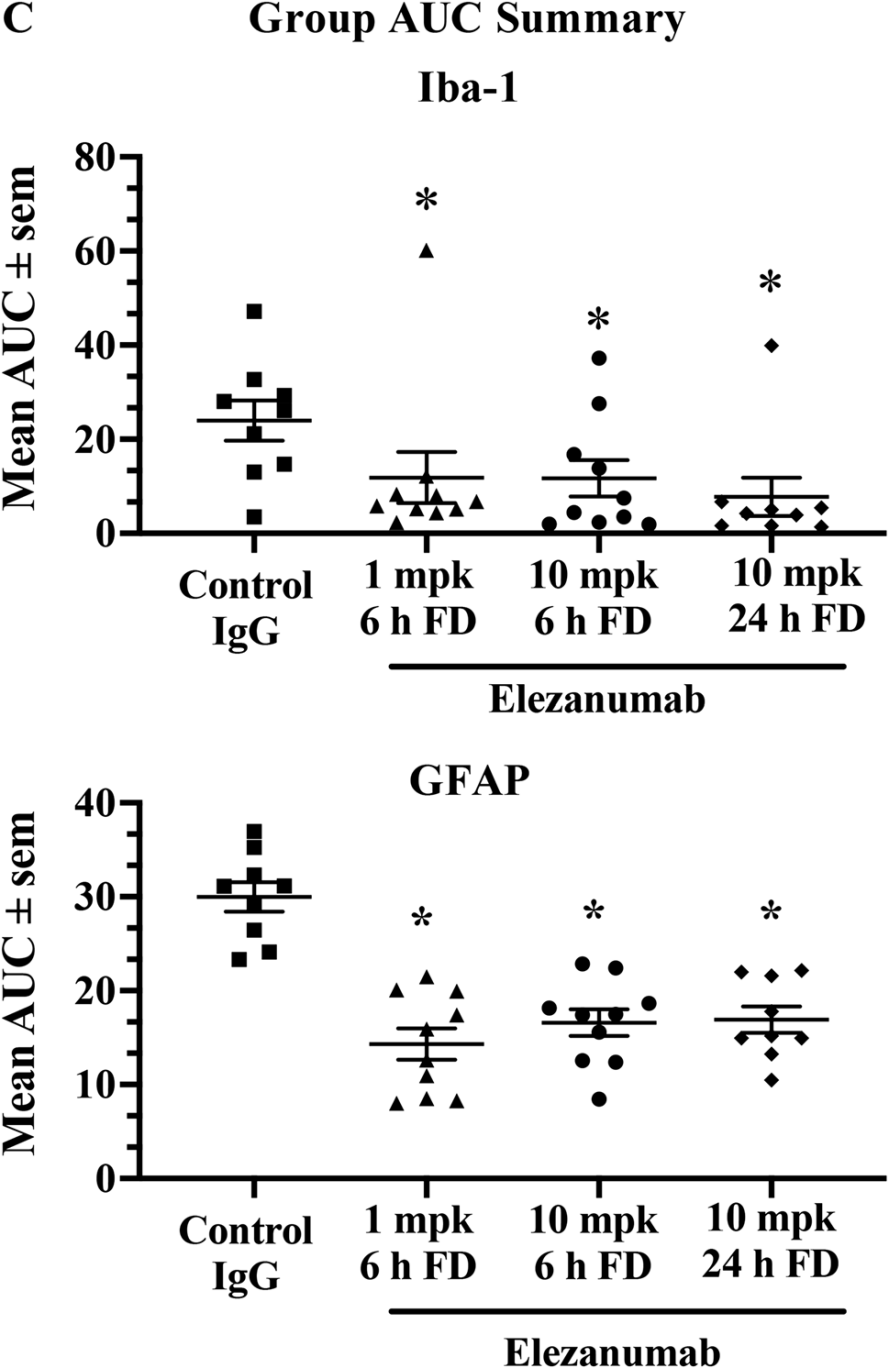
Quantification of Iba-1 and GFAP in elezanumab-treated pMCAO rabbits. **A** Six consecutive Iba-1 stained sections through the midbrain from all control pMCAO animals and from the elezanumab group where the initial dose of 10 mg/kg elezanumab was administered 24 h after injury. Iba-1.^+^ pixels are shown masked in red (see Figure S2 for more details on thresholding). Graphs in panel **B** quantify Iba-1 staining in each slice with respect to distance from the rostral end of the brain. AUCs for each animal are listed in the figure legends. AUC averages for both Iba-1 and GFAP groups in **C** show significant reductions of Iba-1 and GFAP staining in all elezanumab vs. controls animals (*p* < 0.05)

Inhibition of RGMa has been shown to reduce microglial activation and astrogliosis in various pathological conditions [12, 23]; thus, we quantified microglia and astrocyte immunoreactivity using Iba-1 and GFAP, respectively. To help visualize the impact of RGMa neutralization by elezanumab on chronic microglial activation, 6 consecutive sections (from a total of 15–20 sections analyzed per brain), from all IgG control and 24 h FD elezanumab-treated rabbits, are shown in Fig. 6A, where Iba-1 immunoreactivity is depicted in red. With the exception of one animal in the elezanumab group (#586), all other animals had whole brain expression levels of Iba-1 that were near background levels seen in the left hemisphere, which was not directly impacted by the pMCAO procedure. In contrast, a consistent elevation of Iba-1 staining is seen in most of the control animals, localized to 12–15 mm from the rostral aspect of the brain, and primarily confined to the right hemisphere. A similar pattern was observed with GFAP staining (data not shown). The percent of Iba-1^+^ pixels/whole brain tissue area for all groups were then plotted as a function of distance from the rostral end of the brain (Fig. 6B, left-hand graphs). Areas under the curve (AUCs) for whole brain Iba-1 immunoreactivity were calculated for each animal (reported in the figure legends of each graph (Fig. 6B)), with the mean AUCs across groups shown in Fig. 6C for both Iba-1 and GFAP. Analysis of the contralateral left hemisphere illustrates the near-total confinement of Iba-1^+^ staining (and GFAP, data not shown) to the right hemisphere where the occlusion was performed (Fig. 6B, right-hand graphs). Significantly decreased Iba-1 intensity was associated with elezanumab treatment, suggesting attenuation of microglial activation and astrogliosis, even when the first dose was delayed 24 h post pMCAO.

### Lesional Pan-Necrosis Following pMCAO

In study 2, regions of pan-necrosis were evident in the majority of animals in each group. Regardless of treatment, regions presenting with pan-necrosis show loss of GFAP staining with decreased and fragmented NF-200 staining (Figure S4A, B), and Iba-1 staining highlighting foamy round cells consistent with lipid-laden macrophages/microglia (gitter cells; Figure S4A, B). The predominant differences between elezanumab treated and control animals were the size of the regions of pan-necrosis, and the intensity and extent of microglial and astroglial activation as indicated by increased Iba-1 and GFAP staining intensity.

## Discussion

These studies demonstrate that elezanumab administered 6 h after pMCAO enhanced recovery of neuromotor function and suppressed neuroinflammation and astrogliosis, which was also observed when elezanumab was first administered 24 h after pMCAO. These data support a putative cerebroprotective role for RGMa neutralization with elezanumab, potentially with a wider TTI than currently approved standards of care. These findings also confirm previous studies using the elezanumab parental antibody AE12-1Y in a rat embolic pMCAO model with TTI of 2 and 6 h post stroke, showing reduced infarct volume and brain edema and improved functional recovery [13].

Ex vivo MRI/DTI images from the 28-day brains did not clearly identify boundaries of suspected lesions, likely due to the fixation process causing reduced relaxation times for T1, T2, and T2*, and significantly reduced ADC values [24]. Therefore, we used a histological approach to examine the chronic lesions at 28 days. The histopathological changes following pMCAO were consistent with previous studies of experimental brain infarction [25]. Although the pathological changes around regions of pan-necrosis were similar across all groups, all elezanumab-treated animals had significantly fewer activated microglia and astrocytes vs. untreated controls. Elezanumab appears to modulate microglial and astrocyte activation and proliferation following injury through its regulation of RGMa and downstream signaling events [8, 12, 26], and may create a microenvironment more permissive for neuroplasticity and neurorecovery.

In replicate 28-day pMCAO studies described here, elezanumab significantly improved neuromotor function when treatment was first administered 6 h after pMCAO. When treatment was first administered 24 h after pMCAO, although there was no statically significant improvement in neuromotor function, significant suppression of microglial and astrocyte activation was still observed. Similar results were observed in a non-human primate model of acute SCI, where elezanumab significantly improved neuromotor function and increased neuroplasticity in extralesional tissue when administered 3 h after SCI. When the initial dose was delayed to 24 h, elezanumab demonstrated significant effects on neuroplasticity rostral and caudal to the lesion over the 6-month study, but without accompanying improvements in neuromotor function [26]. These data support a unique role for RGMa proximal to the timing of traumatic injury to the CNS.

In addition to measuring functional and histological endpoints, we also confirmed the safety of chronic IV dosing with elezanumab in this pMCAO model. Our data also demonstrate that elezanumab distributes across the blood–brain barrier to brain regions affected by AIS. We minimized unnecessary handling or procedures that might compromise the primary functional assessments, and incorporated many of the committee recommendations embodied in the translational research guidelines: replicate evaluation of multiple doses, clinically relevant intervention times, clinically acceptable route of administration, embolic vs. transient occlusion, confirmation of occlusion in each enrolled animal, study blinding and randomization, statistically powered group sizes, data transparency including mortality and morbidity disclosure, reproducibility, behavioral and histological evidence of efficacy, and extension of initial rat results to a translationally validated experimental model across independent laboratories [17, 18, 27]. Extensions of the present study, including additional groups to evaluate synergy of elezanumab with tPA, evaluation of treatment on acute biomarkers, use of older male and female rabbits, and/or animals with comorbidities, would provide further preclinical insights into the role of RGMa in convergent diseases. However, we focused on adult (not aged) male rabbits with no pre-existing comorbidities to minimize inherent variabilities associated with the aforementioned groups, and importantly, reduce the overall use of a higher species such as the rabbit for translational decision-making.

In summary, these studies provide robust, reproducible, neuromotor, and histological evidence that neutralization of RGMa using elezanumab improves functional recovery and reduces neuroinflammation in an established experimental animal model of AIS. Elezanumab is currently being evaluated in 52-week Ph2 clinical studies for acute ischemic stroke (NCT04309474), as well as acute spinal cord injury (NCT04295538).

### Supplementary Information

Below is the link to the electronic supplementary material.

Supplementary file1 (DOCX 4840 KB)

## Abbreviations

RGMa: Repulsive guidance molecule A
TTI: Time to intervention
pMCAO: Permanent middle cerebral artery occlusion
AIS: Acute ischemic stroke
FD: First/loading dose
MD: Maintenance dose
GFAP: Glial fibrillary acidic protein
NF-L: Neurofilament light chain
IBA: Ionized calcium-binding adapter molecule 1
tPA: Tissue plasminogen activator
MRI: Magnetic resonance imaging
FA: Fractional anisotropy
MTR: Magnetization transfer ratio
ADC: Apparent diffusion coefficient
CBF: Cerebral blood flow
