# Supplementary material for: Neutralizing RGMa with Elezanumab Promotes Cerebroprotection and Recovery in Rabbit Middle Cerebral Artery Occlusion
Source: Transl Stroke Res. 2023 Jun 16;15(4):805–17. doi: 10.1007/s12975-023-01164-2 (PMC11226526; doi:10.1007/s12975-023-01164-2)
Supplement: Supplementary file 1 — Supplementary file1 (DOCX 4840 KB) [file 12975_2023_1164_MOESM1_ESM.docx]

**Supplemental Information**

- Animal Welfare
- Morbidity and Mortality
- NFl Assay
- Antibody characterization and formulation
- Elezanumab and control antibody quantification
- Animal housing
- Rabbit pMCAO surgical procedure
- Histopathology
- Immunohistochemistry
- Longitudinal, non-interventional rabbit pMCAO study
  - Methods
  - Results
- Supplemental Figures and Tables
  - Table S1
  - Figure S1
  - Figure S2
  - Figure S3
  - Figure S4

Animal Welfare

Animal handling followed the guidelines of the National Institute of Health (NIH) and the Association for Assessment and Accreditation of Laboratory Animal Care (AAALAC). Pharmaseed preclinical studies were conducted at Ness-Ziona, Israel. The facility is inspected by the Israel GLP compliance monitoring unit and is accredited by the Israel Laboratory Accreditation Authority (ISRAC) to conduct preclinical studies in compliance with the Organization for Economic Co-operation and Development principles of GLP. These studies were performed in compliance with "The Israel Animal Welfare Act" and followed "The Israel Board for Animal Experiments" approval. All animals were examined by the attending veterinarian to determine fitness prior to study initiation and were inspected twice daily for mortality and any signs of morbidity during the studies.

Morbidity and Mortality

In Study 1, one rabbit died within two h after embolization; no other death or adverse events occurred. In Study 2, eight animals died during the study. Two animals died during or shortly after occlusion, but before the treatment was initiated. Six animals were found dead in their cages within 2-4 days after surgery: 2 rabbits from the elezanumab 10 mg/kg administered at 24 h post-pMCAO; one from the IgG control group on day 2; one rabbit from the IgG control group and one rabbit from the 1 mg/kg elezanumab group on day 3, and one rabbit from the 1 mg/kg elezanumab group on day 4. No other observations of morbidity or mortality were noted.

NFl Assay

The following antibodies were used at a 1:7.5 dilution: NF-L: UD1 for capture and UD2 for detection (Uman Diagnostics). Samples were diluted in standard diluent prior to a 2 h incubation with the capture antibody beads (SMC Capture Reagent Labeling Kit, Merck), and for 1 h with the detection antibody (SMC Detection Reagent Labeling Kit, Merck) at 25°C. All in-between washing steps were performed using an automated plate washer (Tecan) using wash buffer with Proclin (Sigma/Aldrich). Prior to elution, beads were transferred to a new 96 well plate using the Viaflo96 pipetting robot (Integra) to minimize background signals. Elution of detection antibodies from the beads was done with Elution Buffer B for 20 minutes at 25°C. To increase robustness, neutralization and transfer of the elute to a 384 well scanning plate was done in a single tip using the Viaflo96 pipetting robot. Detection of signals was performed using the Erenna (Singulex) platform but could be performed with slight adaptations also on the next generation device SMCxPro (Merck).

Antibody characterizations and formulation

The human anti-RGMa antibody elezanumab (also known as AE12-1Y-QL or ABT-555; AbbVie, North Chicago, IL), is an RGMa-specific human monoclonal antibody (mAb) with no cross-reactivity to RGMa family members RGMb and RGMc.[1] Elezanumab binds human, cynomolgus monkey, and rat RGMa with comparable binding affinity.[1] The binding affinity of elezanumab on recombinant rabbit RGMa is the same as that on rat RGMa generated recombinantly in HEK293 cells[1] since the rabbit RGMa amino acid sequences are identical to rat sequences (unpublished data). The control human IgG used is an anti-tetanus toxin mAb with similar isotype as elezanumab. Stock concentrations of elezanumab (104 mg/ml) were prepared at AbbVie, North Chicago, IL, USA, and the IgG control (41 mg/ml) was prepared at Abbvie, Ludwigshafen, Germany. Both antibodies were used as single lots, and formulated in a 30 mM histidine, 8% sucrose, 0.02% Tween 80, pH 6.0 buffer. All materials were sterile, tested negative for endotoxin (< 0.05 EU/mg), and screened for viral DNAs using PCR. The potency of elezanumab was confirmed using an RGMa reporter gene assay (IC_50_= 97 ± 19 pM). All materials were stored at -80^o^C as individual dosing aliquots to minimize potential freeze/thaw issues.

Elezanumab and control antibody quantification

For the first study (Figure 1A), serum elezanumab concentrations were measured using an electrochemiluminescence (Meso Scale Discovery; MSD) method. Biotinylated human RGMa was used for capture and a SULFO-TAGTM labelled human specific antibody, produced at AbbVie, for detection. A five-parameter logistic model was used for calibration curve fitting for the MSD assay and the lower limit of quantitation (LLOQ) was 156 ng/ml. For all other studies (Figures 1B, 2B, 3B), elezanumab and control antibody concentrations were determined via LC-MS (liquid chromatography-mass spectrometry) methods. Elezanumab and control antibodies were used for calibration standards and quality controls in the respective assays. For brain tissue exposure quantification, PBS-perfused brain tissue was homogenized in radioimmunoprecipitation buffer (RIPA buffer; 10x w/v), and the RIPA soluble fraction was used for analysis. Calibration curves were obtained by spiking the corresponding antibody into rabbit plasma, serum or whole brain lysates. A linear regression model was used to back calculate the measured concentrations of the individual samples for the LC-MS assays, where the LLOQ was 500 ng/mL or ng/g, respectively.

Animal Housing

Male New Zealand White rabbits, weighing 2.8–3.6 kg at study initiation were sourced from Envigo RMS Ltd, Israel. Animals were housed under standard laboratory conditions, with air conditioned and filtered (HEPA F6/6) fresh air (minimum of 15 air changes/h). Animals were housed in a climate-controlled environment with temperatures ranging between 18 and 24°C, and with a relative humidity between 30 and 70% with 12 h light/12 h dark light cycles. Rabbits were single housed in an enriched environment, including hay for their comfort and as a food supplement. Animals were fed a commercial rabbit diet (Teklab Doe Rabbit Diet cat#: 7078S) *ad libitum,* and had free access to standard tap drinking water obtained from the municipality supply.

Rabbit permanent embolic middle cerebral artery occlusion (pMCAO) surgical procedure

Rabbits were anesthetized with ketamine/xylazine (35/5 mg/kg, respectively), and body temperature was maintained at 38°C with a heating pad during the operation. A right lateral neck dissection was performed, and branches of the external carotid artery (ECA) were ligated as described by Zhao et al.[2] The facial branch was cannulated in a retrograde fashion (PE50, ID 0.5 mm, OD 0.9 mm; ADInstruments) toward the ostium of the internal carotid artery (ICA). A temporary aneurysm clip was placed on the common carotid artery (CCA) to prevent systemic injection of the clot. A single embolus (5 mm in length, 0.5 mm width, and weighing 4.5–5.5 mg), prepared 24 h before the surgical procedure from the same rabbit, was injected into the cerebral circulation via the ECA catheter and was flushed with 1.5 ml of normal saline. The clot was visually observed to travel up the proximal 10 mm of the ICA under direct visualization by an operating microscope. The catheter was then removed and the ECA was ligated. The CCA clip was removed to restore flow to the ICA.[2] Cerebral blood flow was measured using a transcranial laser doppler before and immediately after the MCAO procedure to confirm occlusion.

MRI Imaging Details

T2-weighted images were acquired with a rapid acquisition and relaxation enhancement (RARE) method having the following parameters: echo time (TE) = 65 ms, repetition time (TR) = 5700, number of averages = 2, number of echo images = 1, in-plane resolution = 0.150 mm^2^, and slice thickness = 0.750 mm without gap for a total of 40 slices. Magnetization transfer images (M_0_ and M_S_) were likewise acquired with a RARE method having the following parameters: echo time (TE) = 12.5 ms, repetition time (TR) = 5000, number of averages = 2, number of echo images = 1, in-plane resolution = 0.150 mm^2^, slice thickness = 0.750 mm without gap for a total of 40 slices, peak amplitude = 15 mT (M_S_ only), and irradiation offset (M_S_ only) = 1500 Hz. Finally, diffusion tensor images were acquired with a single-shell spin-echo method (echo-planar readout) having the following parameters: echo time (TE) = 25 ms, repetition time (TR) = 10000 ms, number of averages = 1, gradient duration = 5 ms, gradient separation = 10 ms, number of diffusion directions = 16, b-value per direction = 1000 s/mm^2^, in-plane resolution = 0.150 mm^2^, and slice thickness = 0.750 mm without gap for a total of 40 slices.

 T2-weighted images and magnetization transfer ratio (MTR) and apparent diffusion coefficient (ADC) parametric maps were generated, preprocessed, and analyzed using VivoQuant 4.0 software (Invicro, Boston, MA).  MTR was defined by the following formula: *M_0_-M_S_/M_0_*

T2-weighted images were preprocessed by reorientation, denoising with an adaptive non-local means algorithm, and masking around the brain. Segmentation proved difficult based on the T2-weighted images, so it was accomplished manually based on areas of hypointensity and hyperintensity on the MTR and ADC parametric maps, respectively.

Histopathology

Following ex vivo imaging, paraformaldehyde-fixed rabbit brains received at NeuroScience Associates (Knoxville, TN) were treated overnight with 20% glycerol and 2% dimethylsulfoxide to prevent freeze-thaw artifacts. The specimens were then embedded in a gelatin matrix using MultiBrain® Technology. The blocks were rapidly frozen, and after curing, were immersed in 2-methylbutane chilled with crushed dry ice and mounted on a freezing stage of an AO 860 sliding microtome. The MultiBrain® blocks were cut at 40 µm through the entire length of the specimen segment and collected sequentially into a series of 36 containers. All containers contained antigen preserve solution (50% PBS pH 7.0, 50% ethylene glycol, 1% polyvinyl pyrrolidone); no sections were discarded. Each section shown is approximately 1.4 mm apart from its adjacent section.

Immunohistochemistry

Free floating sections were processed for immunohistochemistry using 1x Tris buffered saline (TBS) solution, pH 7.6. After a 3% hydrogen peroxide treatment for 30 minutes, sections were permeabilized with 1x TBS, 0.3% Triton X-100 for 30 minutes, washed 3x for 5 minutes in TBS, and then incubated with the primary antibodies described below overnight at room temperature. Following rinses, sections were incubated for 24 h with a biotinylated secondary antibody at room temperature. After further rinses, sections were incubated with avidin-biotin-HRP complex (VECTASTAIN® Elite ABC, Vector, Burlingame, CA) for 1 h at room temperature. The sections were rinsed, and then treated with diaminobenzidine tetrahydrochloride (DAB) with nickel and hydrogen peroxide to create a visible reaction product. Following further rinses, the sections were mounted on gelatin coated glass slides and air dried. The slides were dehydrated by immersing in increasing concentrations of alcohols, cleared in xylene, and cover slipped.

The following antibodies were used: ionized calcium binding adapter molecule 1 (Iba-1) for microglia (Thermoscientific, Cat# PA5-18039, goat host, 1:5,000 dilution; secondary Ab: Vector, Cat# BA-5000, rabbit host, 1:1000 dilution); glial fibrillary acidic protein (GFAP) for astrocytes (EnCor, Cat# CPCA-GFAP, chicken host, 1:50,000 dilution; secondary Ab: anti-chicken biotinylated, Jackson Labs, Cat# 703-065-155, donkey host, 1:1,000 dilution); NFl-200 to identify neurofilament heavy chain (Encor, Cat# CPCA-NF-H, chicken host, 1:50,000 dilution; secondary Ab: Jackson Labs, Cat# 703-065-155, donkey host, 1:1,000 dilution). Thionine-Nissl was used to stain the cell bodies (sections were mounted on gelatin coated glass slides, air dried and carried through the following sequence: 95% ethanol, 95% ethanol/formaldehyde; 95% ethanol, chloroform/ether/absolute ethanol (8:1:1), 95% ethanol; 10% HCl/ethanol, 95% ethanol, 70% ethanol, deionized water, thionine (0.05% thionine/acetate buffer, pH 4.5), deionized water, 70% ethanol, 95% ethanol, acetic acid/ethanol, 95% ethanol, 100% ethanol, 100% ethanol, 1:1 100% ethanol/xylene, xylene, xylene, coverslip). Digitized slides were analyzed using Fiji/ImageJ open software (version 1.53q).

**Longitudinal, non-interventional characterization of the rabbit pMCAO model**

**Methods**

A non-interventional pMCAO study was performed to better understand the pathophysiology in this model during the early stages post injury as well as evaluate the durability of the injury beyond week 4, when the previous drug studies were terminated. All surgical procedures and neuromotor testing were conducted in an identical manner to those previously used. Cerebral blood flow was measured using a transcranial laser doppler before and immediately after the pMCAO procedure. A total of 76 rabbits were utilized and divided into groups of 4 or 10 animals per group. At various time points post pMCAO, animals were bled, euthanized, and brains removed as previously described. Time points included: normal/no injury, sham (6 h post injury), 24, 48, 72 h; 1, 2, 4 and 8 weeks. No drugs or other interventions were administered during the study. Neuromotor testing was conducted on all animals up until their termination date every 2 weeks. Except for one animal which expired 2 h after surgery, no mortalities were noted in any other group. Neuromotor scores at each timepoint: pre-occlusion, and at 24, 48, 72 h, 1, 2, 3, 4 and 8 weeks post occlusion. Scoring was performed by a single trained researcher who performed the previously reported assessments in Studies 1 and 2. After each scoring session, the evaluation sheets were submitted to Pharmaseed's QA Manager for archiving until the end of the study. Three animals died during the study. One rabbit died during surgery, one rabbit shortly after embolization and one rabbit was found dead in its cage on Day 2 after surgery.

**Results**

Figure S3a illustrates that neuromotor dysfunction peaks near 72 h post injury (NMS=5.05 ± 0.2), followed by a small but sustained recovery through 8 weeks with a final NMS of 3.25 ± 1.48. Coincident with peak functional injury, plasma neurofilament light chain levels also peak 72 h after injury (baseline=39 ± 18 pg/ml; 72 h=1,248 ± 132 pg/ml), returning to baseline values after 30 days (Figure S3a). Circulating neutrophils significantly increased from 12.5% of pre-injury total white blood count (WBC) values, to 48.5% 6 h after injury, returning to near baseline levels after 30 days (Figure S3b). Lymphocyte levels dropped significantly from pre-injury values of 80% WBC down to 43% 6 h after stroke, increasing to baseline levels after 30 days (Figure S3C). Monocyte levels rose more slowly from 1.25% WBC to 4.2% by 48 h, declining to 2.1% by day 7, remaining at that level throughout the 8-week study (Figure S3d). Circulating platelets rose significantly from 200,000/µl to 635,000/µl 72 h post stroke, slowly returning to near baseline levels by 60 days (Figure S3E).

Imaging and histopathology: While in vivo MR imaging of pMCAO rabbits was not available for these studies, all brains were preserved (intact) in PFA at study termination, and imaged ex vivo. Following imaging, brains were then submitted for histopathology where stained sections were compared to their corresponding MRI slices.

Histopathology

Figure 5 illustrates the initial appearance of T2 hyperintensities in the right hemisphere within the first 6 h., followed by significant edema and tissue changes by 72 h and 1 week. Six h after occlusion, an infarct zone can be defined by the loss of Iba-1 staining, particularly the loss of perinuclear staining while some distal branches retain staining. Surrounding this zone, microglia have retracted, thicker processes and evidence of membrane ruffling, consistent with classically defined activated microglia.[3] At 24 h, a central zone of microglial necrosis, likely corresponding to pannecrosis, can be defined where Iba-1 staining is limited to fragmented cell debris without retention of perinuclear or branching morphology (24 h top image). Microglial necrosis is not consistently observed across different animals, and these areas are usually small in proportion to the edema zone, consistent with the resistance of microglia to ischemia. Peripheral to zones of microglial necrosis, a zone of microglia possessing only perinuclear staining or simplified processes, often in a tetrahedral pattern is present. These cells are distributed in the same spacing and pattern as normal tissue (24 h bottom image). It is possible to speculate, based on the staining pattern, that microglia transiently lose Iba-1 expression around 6 h, but survive the ischemic stress and resume production of Iba-1 by the 24 h timepoint. At 48 h, perivascular regions in the areas of microglial resuscitation and zone of activated microglia have infiltration of round cells with no processes or a single short blunt process which are most numerous near the vessel and appear to disseminate into ischemic zones, which continue to appear hyperintense on T2 images. From this timepoint forward, Iba-1 staining intensity of activated microglia increases and infiltrating Iba-1 positive cells become more numerous and have coarse branching or amoeboid morphologies, becoming tightly packed over the following weeks. At the margins of zones of pannecrosis, round cells with a foamy appearance and sparse Iba-1 peripheral staining invade in large numbers, increasing from 1 to 4 weeks. These cells are consistent with classic gitter cells. At 8 weeks, liquefaction of the regions of pannecrosis results in decreasing density of gitter cells. Around 1 week after injury, T2 intensity normalizes in areas of cell infiltration and increased density of microglial cells, but proceeds to decrease in areas of liquefaction that develop from 4 to 8 weeks after occlusion.

**Supplemental Figures**

**Fig. S1**


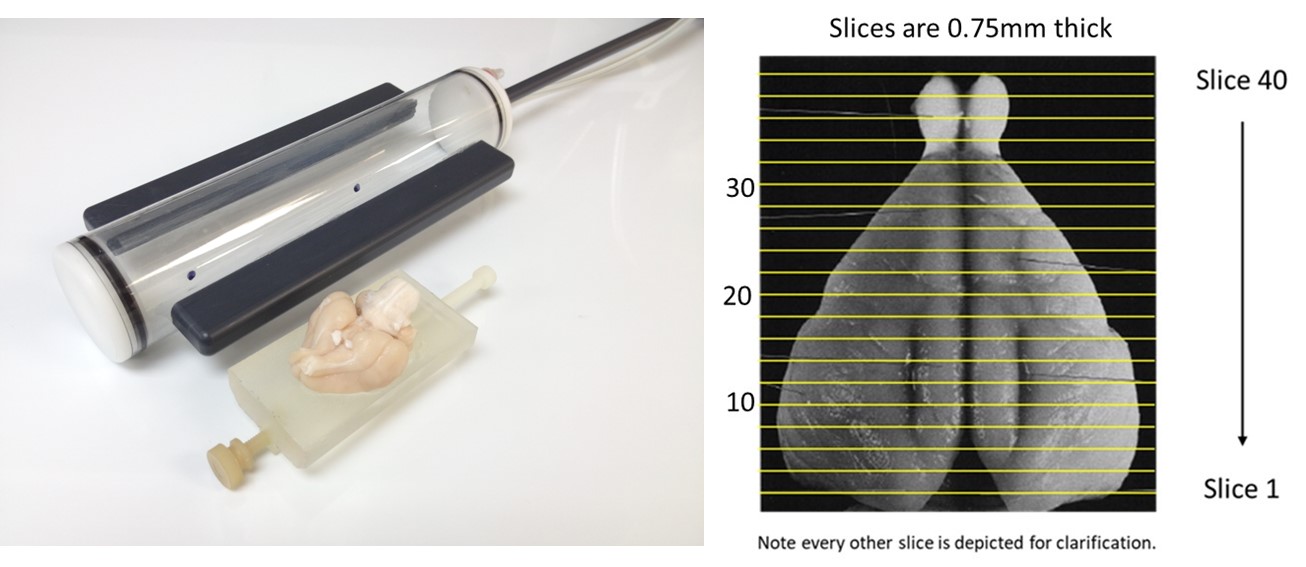


Figure S1: Rabbit brain imaging chamber and brain mold. A custom imaging chamber with a computer aided manufactured rabbit brain mold were designed to secure brains for extended ex vivo imaging sessions. Once the brains were secured in the mold and placed within the chamber, the non-protonated Fluorinert was added to fill the chamber, air evacuated, and then sealed with an airtight cap. Approximately 40 slices, each 0.75 mm thick, were acquired for T1, T2, FA, MTR, and ADC images

**Fig. S2**


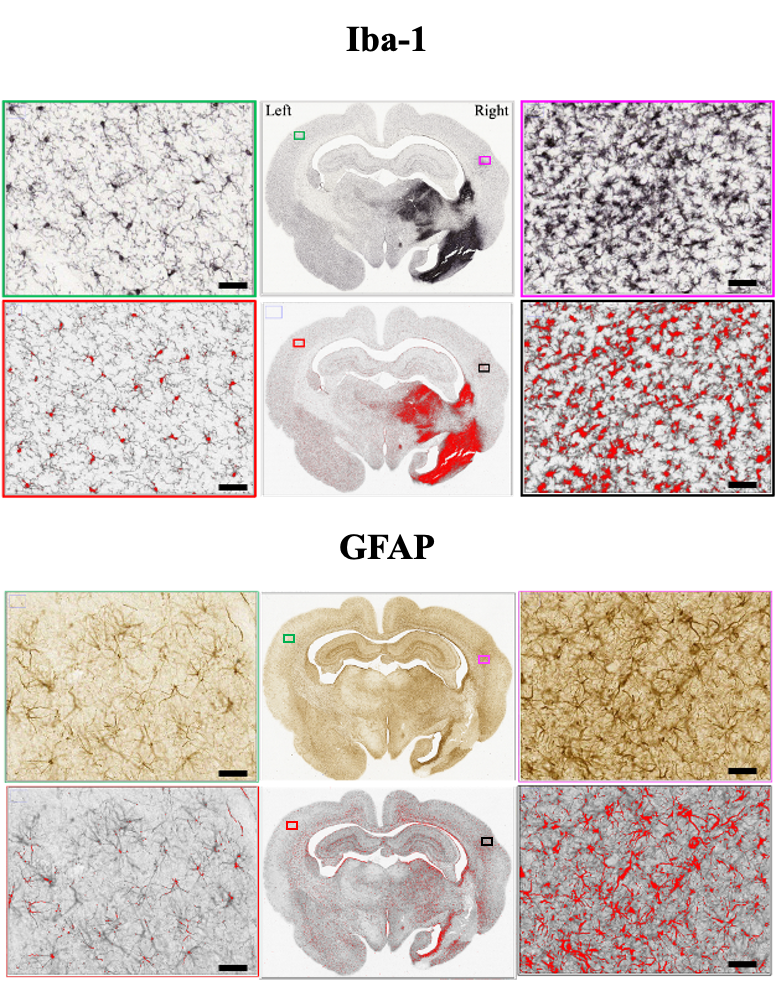


Figure S2: Iba-1 and GFAP staining were quantified by setting baseline threshold intensities using cell bodies in an ‘uninjured’ region from the left hemisphere (identified by boxed areas in the upper left section of the whole slice images for Iba-1 and GFAP, and in magnified panels on the left). Iba-1 and GFAP^+^ positive pixels are shown with red masks in the lower panels for each stain. Boxed regions identified in the upper right section of the whole slice images reveal glial activation consequential to pMCAO, are characterized by greater numbers of cells and larger cell bodies. Following thresholding, Iba-1 or GFAP^+^ pixels were quantified using 15-20 consecutive slices across the brains from each rabbit, and expressed as a percentage of tissue area. It should be noted that these magnified regions significantly underrepresent the extent of glial activation seen in other areas of the whole slice image. Figure S4 provides more details regarding these regions. Scale bars represents 50 µm

**Fig. S3**


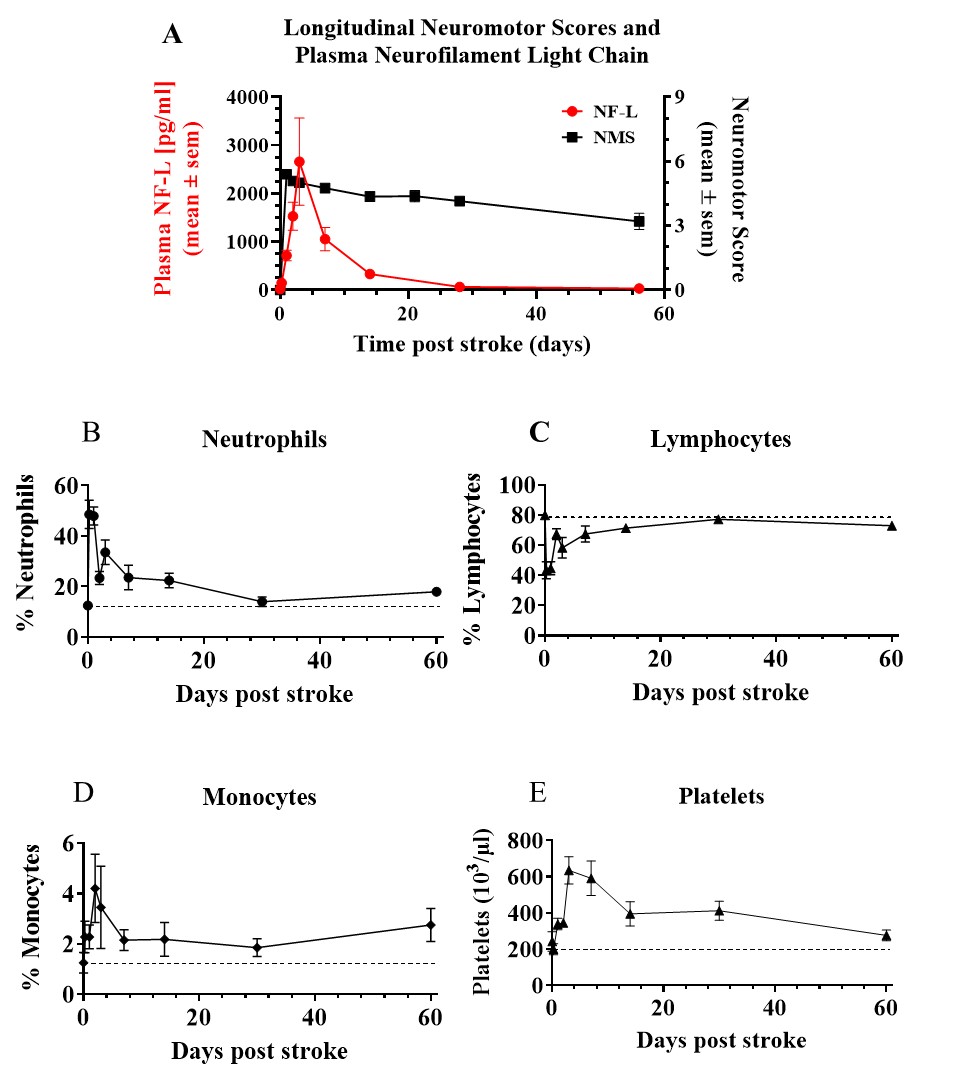


Figure S3: Longitudinal neuromotor, cellular and biochemical changes in the rabbit pMCAO model. A total of 76 rabbits were utilized and divided into groups of 4 or 10 animals per group. At various time points post pMCAO, after neuromotor assessments were completed, animals were bled, euthanized, and brains removed as previously described. Figure S3A shows the average neuromotor scores for untreated animals over 8 weeks following pMCAO surgery. Neuromotor deficits peaked 12-24 h after injury (black line), with minimal recovery over the following 8 weeks. Plasma neurofilament light chain levels rose from ~23 pg/ml at baseline, prior to injury, to 2,659 pg/ml 72 h post-injury, returning to 31 pg/ml by day 56 (Figure S3A; red line). Within 6 h after pMCAO circulating neutrophils increased by over 3x, returning to normal levels by week 4 (Figure S3B). A reverse trend was seen with lymphocytes whose numbers dropped from 80% to 40% six hours after surgery, normalizing by day 30 (Figure S3C). Increases in circulating monocytes and platelets also increased by 48 h after injury, and over the following 60 days, returned to slightly above normal levels (Figures S3D,E). These data collectively emphasize the importance of the first 48-72 h following stroke for treatment of AIS

**Fig. S4**


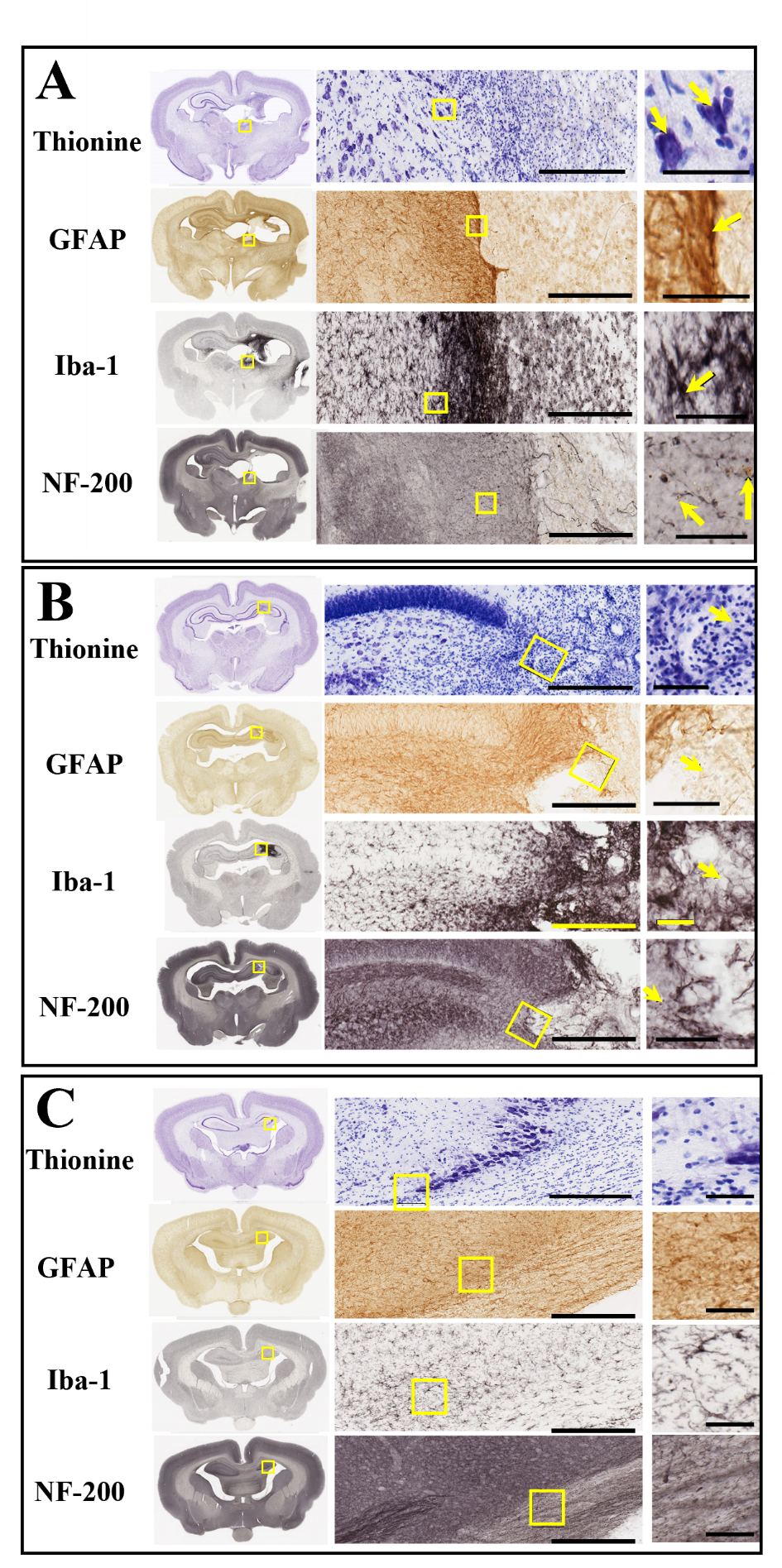


Figure S4: Histopathology. Each panel has images from four stains applied to neighboring sections (thionine, GFAP, Iba-1, NF-200, from top to bottom). Middle images are aligned across stains for an individual animal, to the extent that serial sections could be aligned (scale bar represents 300 um). Right images highlight cellular detail at higher magnification.

Panel A. Control rabbit 30 days after MCAO procedure. Middle image thionine staining demonstrates intact brain tissue on left with marginal region of selective neuronal necrosis and pan-necrosis on right side. Intact neurons appear shrunken at high power (arrows). GFAP staining demarcates the region of pan-necrosis to the right and is bordered by a glial scar (arrow). Iba-1 positive gitter cells exist in region of pan-necrosis. At high power, microglia in region of selective neuronal necrosis have finely branched processes. Some neurites extend into region of pan-necrosis and hemosiderin pigment is evident, suggesting former hemorrhage (arrows). High power scale bars represent 50 µm.

Panel B. Elezanumab-treated rabbit 30 days after MCAO procedure with region of pan-necrosis. Thioinine staining demonstrates abrupt loss of dorsal dentate gyrus neurons (center image) and the hypercellularity associated with the infarcted region. The right thionine figure shows foamy phagocytic cells which are unstained by GFAP (arrows). Iba-1 staining demonstrates staining in the plasma membrane which outlines gitter cells (arrow; the region selected is not contained within the middle image, so it is not outlined). Neurofilament staining shows markedly reduced staining in the area of pan-necrosis and apparent fragmentation of some neurites (arrow). High power scale bars represent 60 µm.

Panel C. Elezanumab-treated rabbit 30 days after MCAO procedure lacking region of pan-necrosis. Thionine staining demonstrates abrupt loss of hippocampal neurons without formation of gitter cells (selective neuronal necrosis). GFAP staining is slightly more intense in area of partial neuronal loss, but a distinct glial scar is not present. In areas of selective neuronal necrosis, Iba-1 staining demonstrates presence of ramified microglia and NF-200 staining shows preservation of neurite processes. High power scale bars represent 50 µm.

[1] L. Huang *et al.*, "Elezanumab, a clinical stage human monoclonal antibody that selectively targets repulsive guidance molecule A to promote neuroregeneration and neuroprotection in neuronal injury and demyelination models," *Neurobiol Dis,* vol. 159, p. 105492, Nov 2021, doi: 10.1016/j.nbd.2021.105492.

[2] B. Q. Zhao, Y. Suzuki, K. Kondo, K. Kawano, Y. Ikeda, and K. Umemura, "Cerebral hemorrhage due to heparin limits its neuroprotective effects: studies in a rabbit model of photothrombotic middle cerebral artery occlusion," *Brain Res,* vol. 902, no. 1, pp. 30-9, May 25 2001, doi: 10.1016/s0006-8993(01)02285-5.

[3] J. Lier, W. J. Streit, and I. Bechmann, "Beyond Activation: Characterizing Microglial Functional Phenotypes," *Cells,* vol. 10, no. 9, Aug 28 2021, doi: 10.3390/cells10092236.
